# Optimizing Colocalized Cell Counting Using Automated and Semiautomated Methods

**DOI:** 10.1007/s12021-025-09723-8

**Published:** 2025-03-21

**Authors:** Hasita V. Nalluri, Shantelle A. Graff, Dragan Maric, John D. Heiss

**Affiliations:** 1 Surgical Neurology Branch, Flow and Imaging Cytometry Core Facility, Bethesda, MD USA; 2 Surgical Neurology Branch, Disorders and Stroke, National Institute of Neurological, National Institutes of Health, Bethesda, MD USA

**Keywords:** Colocalization, Quantification, Tissue

## Abstract

Inflammation within the spinal subarachnoid space leads to arachnoid hypercellularity. Multiplex immunohistochemistry (MP-IHC) enables the quantification of immune cells to assess arachnoid inflammation, but manual counting is time-consuming, impractical for large datasets, and prone to operator bias. Although automated colocalization methods exist, many clinicians prefer manual counting due to challenges with diverse cell morphologies and imperfect colocalization. Object-based colocalization analysis (OBCA) tools address these issues, improving accuracy and efficiency. We evaluated semi-automated and automated OBCA techniques for quantifying colocalized immune cells in human arachnoid tissue sections. Both methods demonstrated sufficient reliability across morphologies (P < 0.0001). While automated counts differed significantly from manual counts, their strong correlation (R^2^ = 0.7764–0.9954) supports their reliability for applications where exact counts are less critical. Additionally, both techniques significantly reduced analysis time compared to manual counting. Our findings support the use of automated and semi-automated colocalization analysis methods in histological samples, particularly as sample size increases.

**Supplementary Information:**

The online version contains supplementary material available at 10.1007/s12021-025-09723-8.

## Introduction

The spatial interactions between different biological structures, broadly referred to as colocalization, are an important area of study for both cell biologists and clinicians. Human tissue, like all biological tissues, is composed of a multitude of diverse cell populations, proteins, and other biochemical structures. Researchers can label specific structures with different fluorescent reporter proteins and visualize their physical interactions with multi-fluorescent imaging. Colocalization is defined as the spatial overlap of two or more biological structures, and colocalization analysis is a common method employed by scientists when analyzing multi-fluorescent imaging data (Bolte & Cordelieres Dec, 2006).

Advances in microscopy and imaging have greatly enhanced our abilities to visualize biological structures and interactions. At the same time, though, imaging data has become increasingly complex and so has its subsequent analysis. Thus, imaging data analysis has become a significant challenge and “bottleneck” in the research process. Given that colocalization analysis is a widely used technique, several programs have been developed to streamline and automate this process (Lunde & Glover, 2020).

Colocalization analysis techniques can be broadly divided into two categories: pixel-based and object-based. Pixel-based methods, like Coloc2 (Rajani et al., 2021), look at each individual pixel across all the channels in a given multi-channel image, comparing fluorescence intensities for that pixel. Colocalization using pixel-based methods can be estimated by calculating the percentage of pixels in an image that are colocalized with one another. This measurement is known as a colocalization coefficient and can be expressed as both a Pearsons’s correlation and a Manders coefficient (Manders et al., Mar 1993; Zinchuk & Grossenbacher-Zinchuk, 2009). Pixel-based methods examine each pixel individually and not as part of a biological structure, spatial exploration of a colocalized signal is not possible (Barlow et al., Dec 2010).

In contrast, object-based colocalization analysis (OBCA) methods allow for spatial exploration because they first identify biological structures as “objects” for further analysis (Gilles et al., 2017). Each channel image in the multi-channel image is segmented to distinguish image background signal from object signal, enabling object identification. Image segmentation is often performed by intensity thresholding, under the assumption that significantly different intensities than the background. Image segmentation tends to be the hardest step of OBCA pipelines because it is hard for segmentation algorithms to capture and account for the variability and complexity of cell morphologies (Meijering, 2012). After segmentation, the center of each object, termed the centroid, is determined based on the object shape and signal. Objects are considered colocalized when their centroids are within a certain distance (Lunde & Glover, 2020).

A key benefit of the centroid object-based approach is that objects do not have to be overlapping to be considered colocalized. For our colocalization analysis in this study, we assessed arachnoid tissue inflammation and hypercellularity by measuring colocalized immune cell counts. In our tissue, as in most human and biological tissue samples, cells that we consider to be “colocalized” co-expressed markers that did not always overlap in space. Colocalized immune cells were defined as expressing a nuclear and cytoplasmic marker which, despite being found in the same cell, are physically not in the exact same space. Hence, our analysis involved object-based colocalization techniques.

Most researchers and clinicians’ default to the manual colocalized cell counting approach despite the development of automated techniques, mainly because automated algorithms are not always biologically accurate. Cells and biochemical structures have diverse and complex morphologies. Even with state-of-the-art fluorescent labeling and imaging, it is very difficult for an automated algorithm to segment and identify objects of such complex nature. Manual analysis is considered the “gold standard” because a researcher, who understands these complex morphologies, can accurately identify objects that an automated program would miss. Semi-automated colocalization techniques have also been developed, which combine automated analysis with manual user intervention. Some argue that, while automated OBCA algorithms are prone to errors, a semi-automated approach is a viable alternative to purely manual colocalization analysis.

While the manual approach is considered the gold standard, it is impractical for large data sets and can be tedious. Additionally, manual counting introduces operator error and bias. Ultimately, there is a need for a biologically accurate, automated OBCA program. To this end, we compared semi-automated and automated colocalization analysis techniques to see if they are sufficiently reliable to aid in clinical decision-making and research. Our analysis was conducted in human arachnoid tissue samples, an ideal sample because of its diverse and complex cell populations.

## Methods

### Image Sample Acquisition

#### Tissue Acquisition

The data presented in this study was obtained from participants enrolled in NIH protocol 10N0143, Natural History Study of Patients with Syringomyelia. Consent was obtained from all patients. Nine patients from this study underwent neurosurgical treatment for syringomyelia associated with arachnoiditis. Surgical specimens of arachnoid tissue were collected from these patients during surgery for treatment of syringomyelia and/or associated arachnoiditis and embedded in paraffin for future tissue processing.

#### Image Acquisition

 Tissue samples were later deparaffinized and underwent multiplex immunohistochemical (MP-IHC) staining for visualization of nuclear (DAPI) and immune cell (CD4, CD8, CD20, CD68, IBA1) markers. We followed a well-accepted MP-IHC method (Riggle et al., 2020). Briefly, the arachnoid tissue sections were de-paraffinized, then exposed to the following primary antibodies: Goat IgG anti-CD4 (R&D Systems) to identify helper T-cells, Mouse IgG2b anti-CD8 (Thermo Fisher Scientific) to identify cytotoxic T-cells, Mouse IgG2a anti-CD20 (Thermo Fisher Scientific) to identify B-cells, and Mouse IgG3 anti-CD68 (Thermo Fisher Scientific) in combination with Chicken IgY anti-IBA1 (Cedarlane Labs) to identify infiltrating macrophages. After washing off excess primary antibodies, sections were incubated with the appropriately cross-absorbed secondary antibodies, facilitating their binding with the species-specific epitopes of the primary antibodies and subsequent visualization of the markers. These secondary antibodies (purchased from either Thermo Fisher Scientific or Jackson ImmunoResearch) were conjugated to one of the following spectrally compatible fluorophores: Alexa Flour 488, Alexa Flour 647, Alexa Flour 594, Alexa Flour 546, Alexa Flour 790, Alexa Flour 680, or IR800CW. The image samples analyzed in this study were acquired from whole images of arachnoid tissue sections. The initial tissue samples were imaged using the Axio Imager.Z2 slide scanning florescence microscope (Zeiss) equipped with a × 20/0.8 Plan-Apochromat (Phase-2) non-immersion objective (Zeiss), a high-resolution ORCA-Flash 4.0 sCMOS digital camera (Hamamatsu), a 200W X-Cite 200DC broad band lamp source (Excelitas Technologies), and filter sets (Semrock) customized to detect different fluorophores. Image tiles (600 × 600 um viewing area) were individually captured at 0.325 micron/pixel spatial resolution, and the tiles were stitched into whole specimen images using the ZEN 2 image acquisition and analysis software program (Zeiss). Pseudocolored stitched images were overlaid in Imaris 9.2.1 (Bitplane) as individual layers to create multicolored merged composites.

To standardize the pixel dimensions of each image sample for counting, sections of each stained tissue image were selected to cover an area of 25,000,000 square pixels. For larger tissue images, multiple non-overlapping sections were selected as different samples (from one patient specimen). Image sections were chosen such that areas with zero, low, medium, and high colocalized cell populations were included in the counting analysis. 27 arachnoid tissue image samples were collected from 9 patients for analysis.

**Fig. 1:**
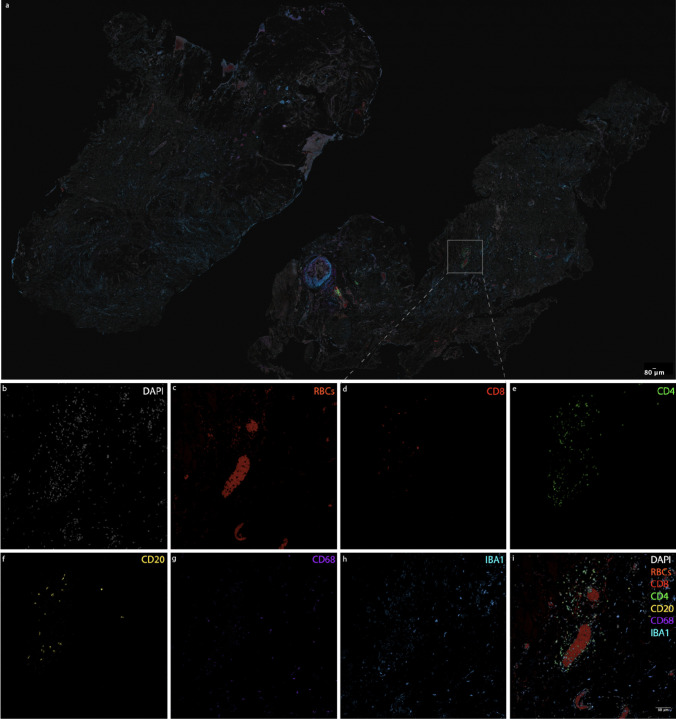
Representative images of nuclear (DAPI), red blood cell (RBC), and immune cell markers in a tissue sample following multiplex immunohistochemistry (MP-IHC). (a) Overview image of an arachnoid tissue section with an area of 25.4 mm^2^, illustrating the spatial distribution and colocalization of the marker analyzed in this study. Panels (b–i) show higher magnification views of the same tissue, highlighting specific marker expression. The scale bar in panel (a) applies only to panel (a), while the scale bar in panel (i) applies to panels (b–i). Both scale bars represent 80 µm

The tissue in each image sample had been stained for the nuclear marker DAPI as well as several immune cell markers: CD4, CD8, CD20, CD68, IBA1. A colocalized cell was defined as a cell that was co-stained for the nuclear DAPI marker and an immune cell marker. Colocalized cell counts were conducted for each immune cell marker type individually; cells that were colocalized for DAPI, CD68, and IBA1 were also quantified. Two independent observers performed the colocalized cell counts on the tissue samples, using a manual and semi-automated colocalized cell counting method. The automated method did not require user input (Fig 1).

##### Image Pre-Processing

**Fig. 2:**
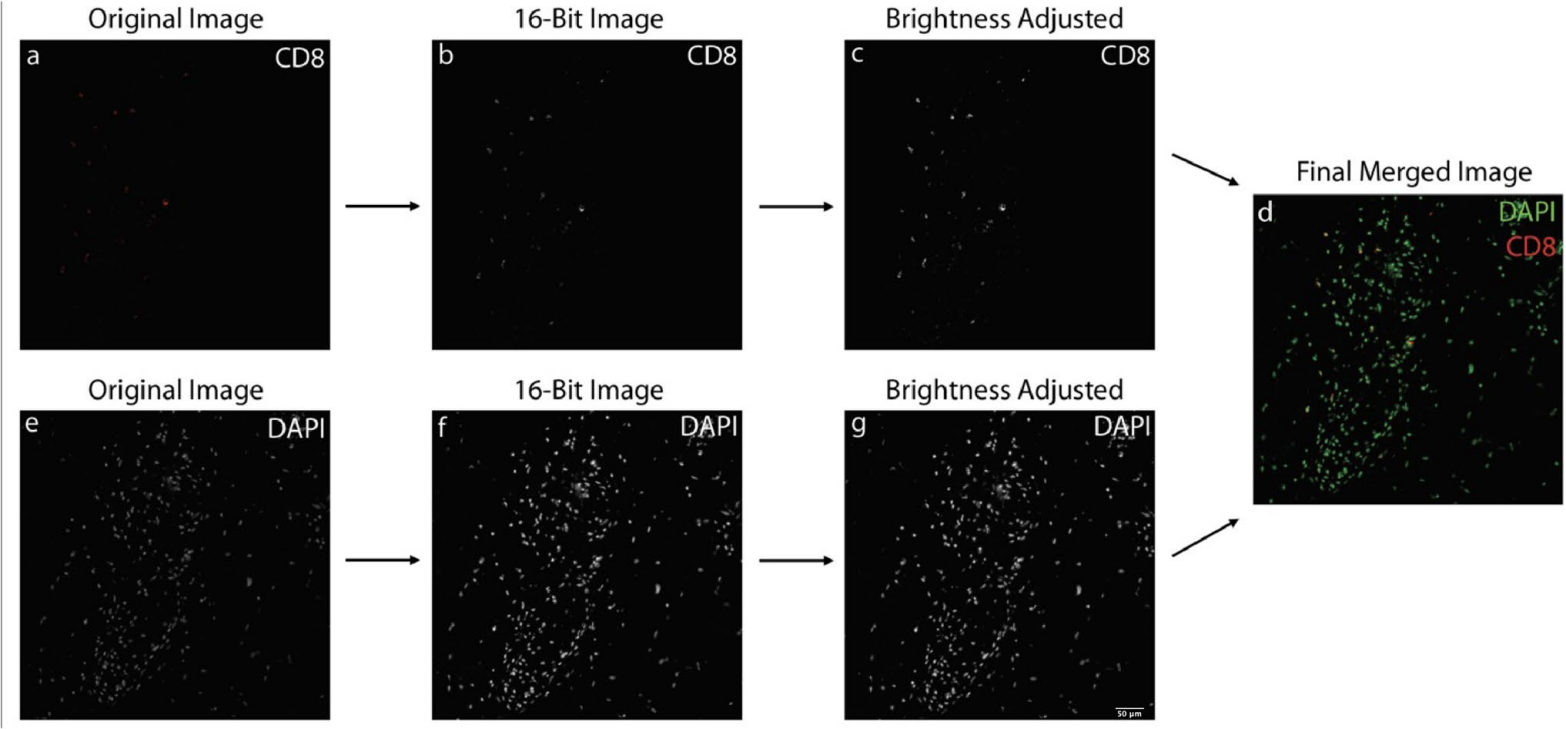
Flowchart illustrating the image preprocessing steps. The original images (a and e) are converted into 16-bit black-and-white images (b and f), followed by adjustments to brightness and contrast (c and g). These preprocessed images are then merged to create a composite image, with DAPI (nuclear stain) displayed in green and the immune cell marker in red (d). The scale bar in panel (g) is equal to 50 µm and applies to panels (a-d)

For the manual and semi-automated counting methods, images were pre-processed to streamline counting and, if necessary, improve visibility of the staining. For each immune cell marker, composite images of the sample tissue stained with the nuclear marker and respective immune marker were prepared from the individual channel images. Each composite image was prepared by first opening the nuclear and immune marker channel images on ImageJ. Each channel image was converted into a 16-bit image; if the brightness of the staining was too low, the Adjust Brightness and Contrast feature on ImageJ was used to increase brightness and contrast. The "Merge Channels" ImageJ function was used to merge the nuclear and immune marker composite image. In each composite image, the nuclear channel (DAPI) image was the green channel, and the respective immune marker channel image was the red channel. For the analysis of DAPI/CD68/IBA1 colocalized cells, a composite image was created from the three channels, with the green channel displaying the DAPI staining; the red channel displaying the CD68 staining; and the blue channel displaying the IBA1 staining. Composite images were prepared for all 27 samples, and each immune cell marker for those samples (CD4, CD8, CD20, CD68, IBA1). Each observer independently pre-processed the image samples. To prevent discrepancy in counts due to different image pre-processing, each observer used the same composite image for the manual and semi-automated counting methods (Fig 2).

##### Manual Counting Method

The multi-point tool on ImageJ was used to perform the manual colocalized cell counts. For each composite image containing a specific immune and nuclear marker, the observer zoomed in until individual cells were clearly distinguishable. The observer then identified cells co-stained with DAPI and immune markers, manually marking each identified cell with a multi-point. After reviewing the entire image sample, the observer recorded the total number of multi-points as the count of colocalized cells for that particular immune marker. This process was repeated for DAPI/CD68/IBA1 colocalized cells, except the observer was looking for cells co-stained for all three markers.

##### Semi-Automated Counting Method

The Colocalization Object Counter ImageJ plugin, developed by Lunde and Glover (Lunde & Glover, 2020) was used to perform the semi-automated colocalized cell counts. Access to the plugin can be found here: https://sites.imagej.net/ObjectColocalizationPlugins/. This ImageJ plugin allows for an automated centroid-based approach to colocalized cell counting, with manual verification and correction. For each sample, the plugin and composite image were loaded into ImageJ. The nuclear channel (green) was opened first in the composite image, and the "Find 2D maxima (as multipoints)" plugin tool was clicked to automatically mark all the nuclei in the image sample with multipoints. If nuclei were overcounted or background noise was detected as counts, the "Pre blur radius" and "Noise tolerance" parameters were increased accordingly until nuclei were no longer being overcounted. With category 1 checked, the "Convert multipoint to overlays" tool was then clicked to save the marked nuclear multipoints as overlays for cells.

Next, the immune channel (red) was opened in the composite image. "Find 2D maxima (as multipoints)" was used to automatically mark all the corresponding immune cells in the image sample with multipoints. As described above, the "Pre blur radius" and "Noise tolerance" parameters were adjusted accordingly until immune cells were being properly counted. After the automatic immune cell detection, the observer then reviewed the image sample to manually add or remove any marked immune cells as needed. The checked category on the plugin tool was switched from 1 to 2. With category 2 checked, the "Convert multipoint to overlays" tool was then clicked to save the marked immune multipoints under category 2 cells.

By clicking the "Count information" button on the plugin, the observer can see the number of cells marked under category 1 (nuclear) and category 2 (immune). The plugin also outputs the number of colocalized cells, marked under category 1 and 2 (termed category 12). The observer recorded the number of category 12 cells as the count of colocalized cells for that particular immune marker.

**Fig. 3:**
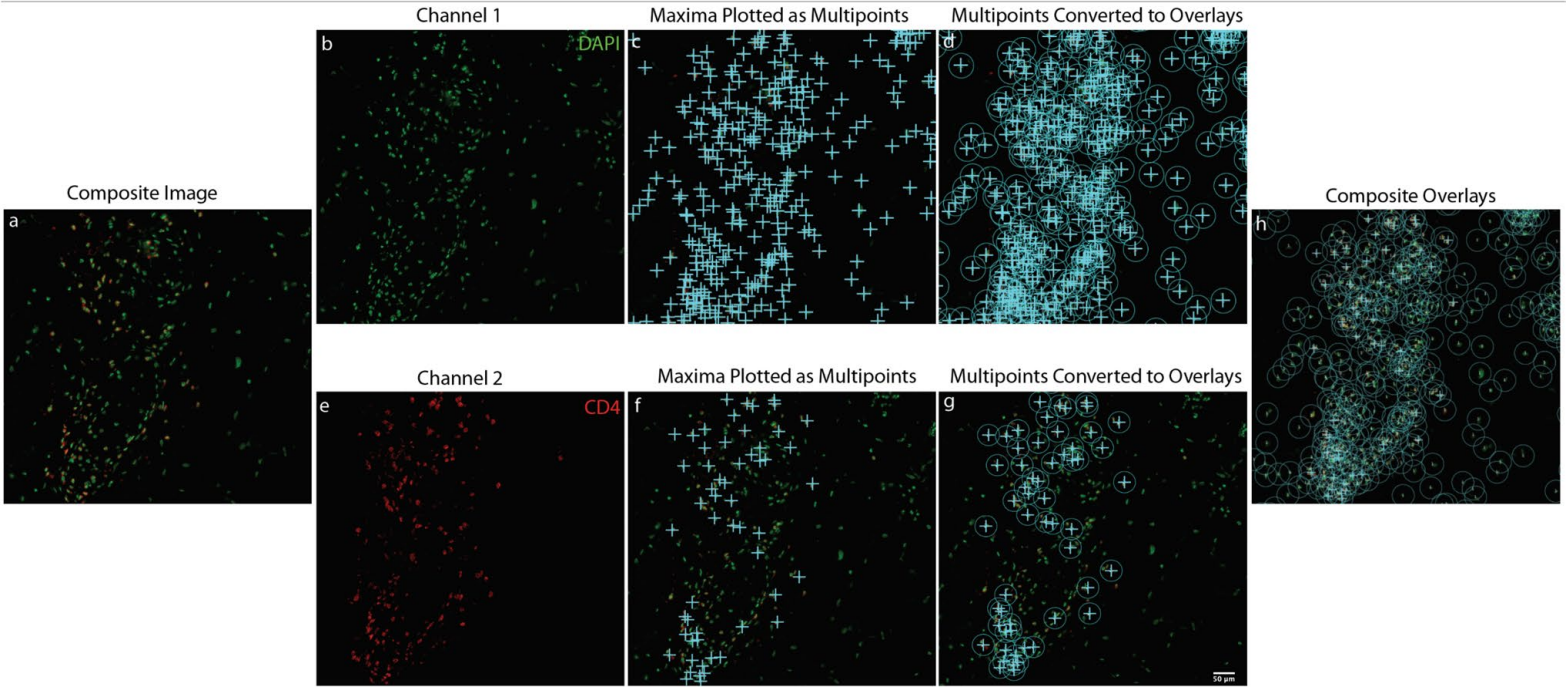
Analysis of a two-channel image containing DAPI (green) and an immune cell marker CD4 (red) using the semi-automatic counting method. These images depict cells with an ‘easy-to-count’ morphology. Panel (a) shows the composite image displaying both channels, while panels (b) and (e) display the individual green (DAPI-positive nuclei) and red (IBA1-positive cells) channels, respectively. Panels (c) and (f) illustrate the identification and plotting of 2D maxima in the green and red channels. In panels (d) and (g), the plotted maxima are converted into overlays of a defined radius, with panel (h) specifically showing the merging of overlays from both channels, where overlapping regions between channel 1 (DAPI, green) and channel 2 (CD4, red) are counted as colocalized objects. The scale bar in panel (g) is equal to 50 µm and applies to all panels

**Fig. 4:**
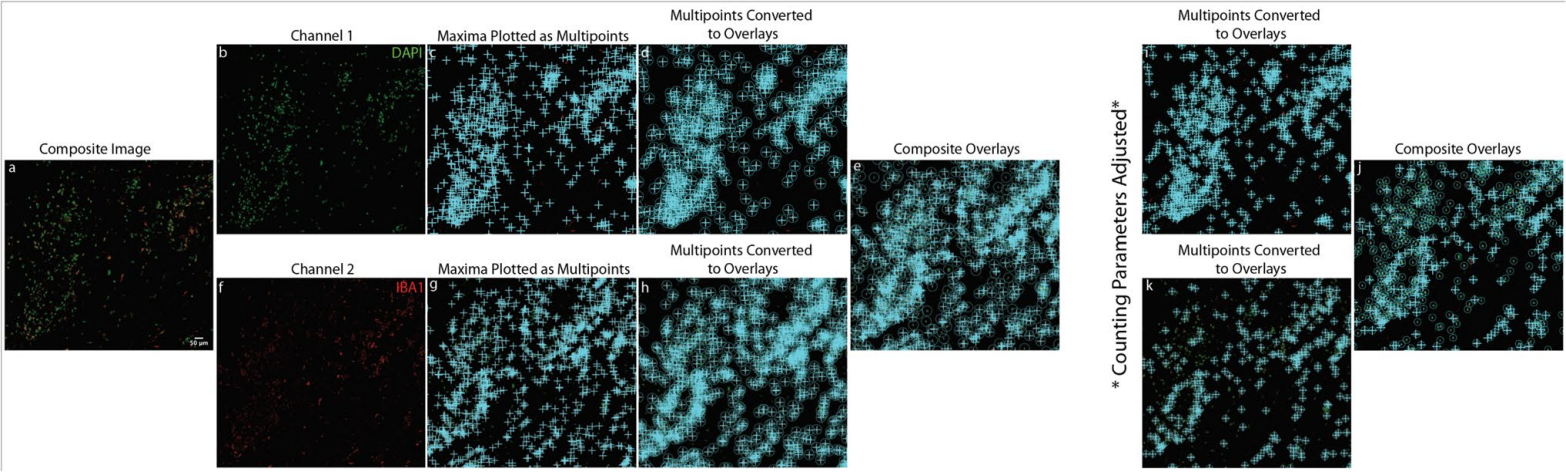
Analysis of a two-channel image containing DAPI (green) and the immune cell marker IBA1 (red) using a semi-automatic counting method. The images depict cells with a "hard-to-count" morphology. Panel (a) shows the composite image displaying both channels, while panels (b) and (f) display the individual green (DAPI-positive nuclei) and red (IBA1-positive cells) channels, respectively. Panels (c) and (g) illustrate the identification and plotting of 2D maxima in the green and red channels. In panels (d) and (h), the plotted maxima are converted into overlays of a defined radius, with panel (h) specifically showing the merging of overlays from both channels. Overlapping regions between the DAPI and IBA1 channels are counted as colocalized objects. Due to the irregular and fragmented structure of IBA1-positive cells, initial counts were overestimated, as seen in panel (e). To account for this "hard-to-count" morphology, counting parameters were adjusted by setting the pre-blur radius to 1 and reducing the overlay radii from 50 to 30. These adjustments improved the accuracy of maxima detection in the green (DAPI) channel (i) and red (IBA1) channel (k), resulting in a substantial reduction in overcounting. The final refined counts are shown in panel (j). The scale bar in panel (a) is equal to 50 µm and applies to all panels

This process was repeated for all immune markers across all samples. For DAPI/CD68/IBA1 colocalized cells, the process was the same except an additional category (category 3) was included. As described above, the DAPI multipoints were in category 1 and the CD68 (an immune marker) multipoints were in category 2. The IBA1 multipoints were marked under category 3, and the colocalized cell count was the number of category 123 cells for the image (Figs. 3 and 4).

##### Automated Counting Method

A custom object-based colocalization analysis pipeline was run on the widely used CellProfiler software to perform the automated colocalized cell counts (Fonseca & Rosa, 2025; Jones et al., 2009; Zhang et al., Feb 2018). The CellProfiler software is available for download here: https://cellprofiler.org/releases.

The pipeline consisted of successively performing CorrectIlluminationCalculation (smoothing method = Fit Polynomial) on both channel images, CorrectIlluminationApply, IdentifyPrimaryObjects (threshold strategy = global; thresholding method = Otsu; Three classes thresholding) on both images, RelateObjects, ExpandOrShrinkObjects (shrink objects to a point) on both images, ExpandOrShrinkObjects (expand objects 5 pixels) on both images, RelateObjects, ExportToSpreadsheet. Objects within 5 pixels of each other between the 2 channels were considered colocalized by the algorithm.

Images were not pre-processed for the automated counting method. To perform the counting analysis, un-processed nuclear and immune marker channel images were inputted into the CellProfiler software. After the analysis was complete, the pipeline exported a spreadsheet of results, including the number of colocalized objects. The number of colocalized cells was the number of objects counted by the pipeline for that particular sample and marker.

**Fig. 5:**
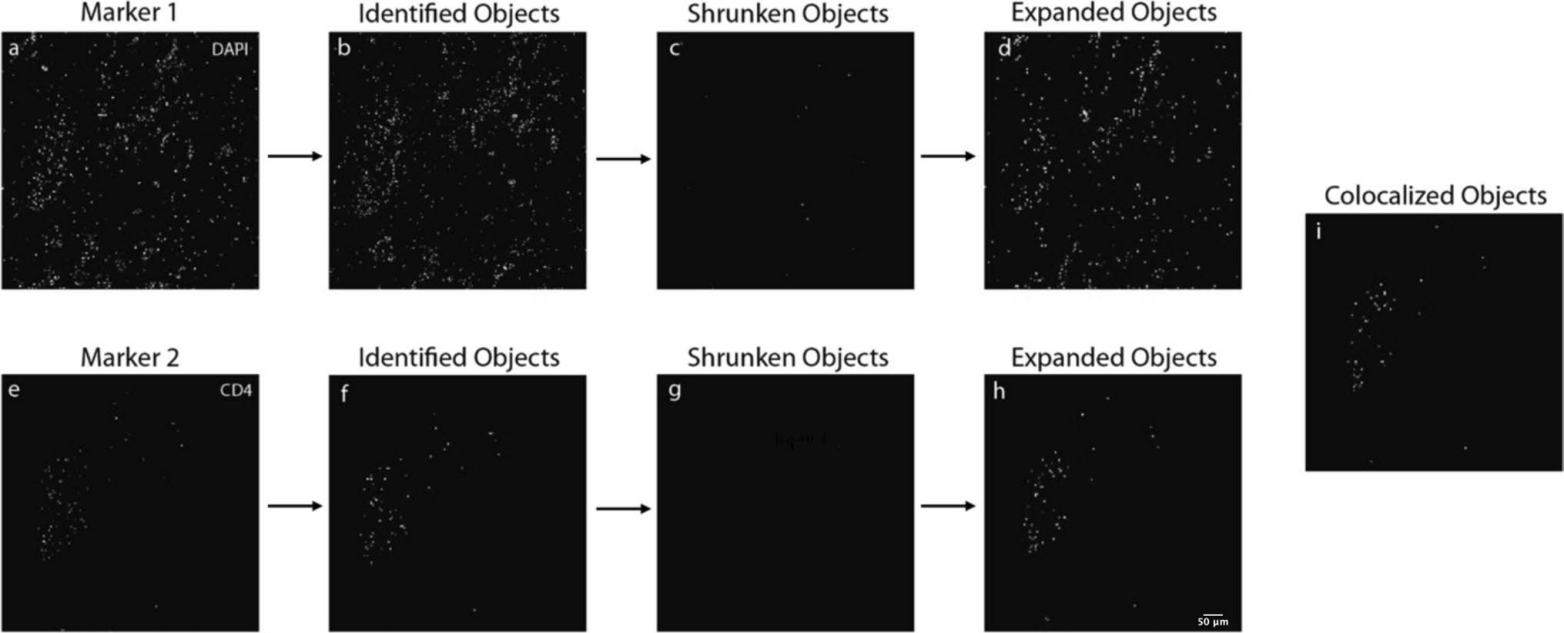
Schematic representation of the automated object-based colocalization pipeline applied to a sample image containing two distinct marker channels. The pipeline processes two separate channels (a and e) to identify and quantify colocalized objects (i), enabling the analysis of overlapping signals from the two markers. The scale bar in panel (h) is equal to 50 um and applies to panels (a-i)

The same pipeline was run for the DAPI/CD68/IBA1 colocalized cells, with the addition of a segmentation and object identification module. The pipeline consisted of successively performing the same pipeline as described above, with the additional steps ClassifyObjects, FilterObjects, IdentifyPrimaryObjects on the third channel image, RelateObjects, ExpandOrShrinkObjects (shrink objects to a point) on both images, ExpandOrShrinkObjects (expand objects 5 pixels) on both images, RelateObjects, ExportToSpreadsheet. These additional steps accounted for the extra channel to be analyzed (Fig 5).

##### Time Calculations

During the counting process for the manual and semi-automated methods, each observer recorded the time it took them to collect the colocalized cell counts from opening the image to saving the colocalized cell count number. Both the manual and semi-automated counting methods required image pre-processing. The time to pre-process each image sample was measured and added to the time calculation accordingly.

Initial setup of the automated pipeline took 15 min. As the images did not require pre-processing, the time it took to count each image sample was the processing time of the software. For each immune marker, the pipeline was run for all samples at the same time; the time to analyze each image was recorded by the software and outputted in the spreadsheet of results.

##### Statistical Analysis

A correlation analysis was conducted between semi-automated vs. manual and automated vs. manual counts to determine how accurate the semi-automated and automated methods were to the "standard" manual counting method. Paired t-tests were used to compare the absolute colocalized cell counts between semi-automated and automated counts to manual counts. The average count values between the two observers were used for all the listed statistical analyses. To evaluate inter-operator variability, paired t-tests were conducted for the measured counts between both observers. All statistical analysis was performed in Prism 10.

## Results

Manual, semi-automated, and automated colocalized cell counting was performed on arachnoid tissue containing various cell types and morphologies including helper T-cells, cytotoxic T-cells, B-lymphocytes, and macrophages. Since manual counting is currently considered the “gold standard” method for colocalized cell counting, semi-automated and automated counts were compared to manual counts.

**Fig. 6:**
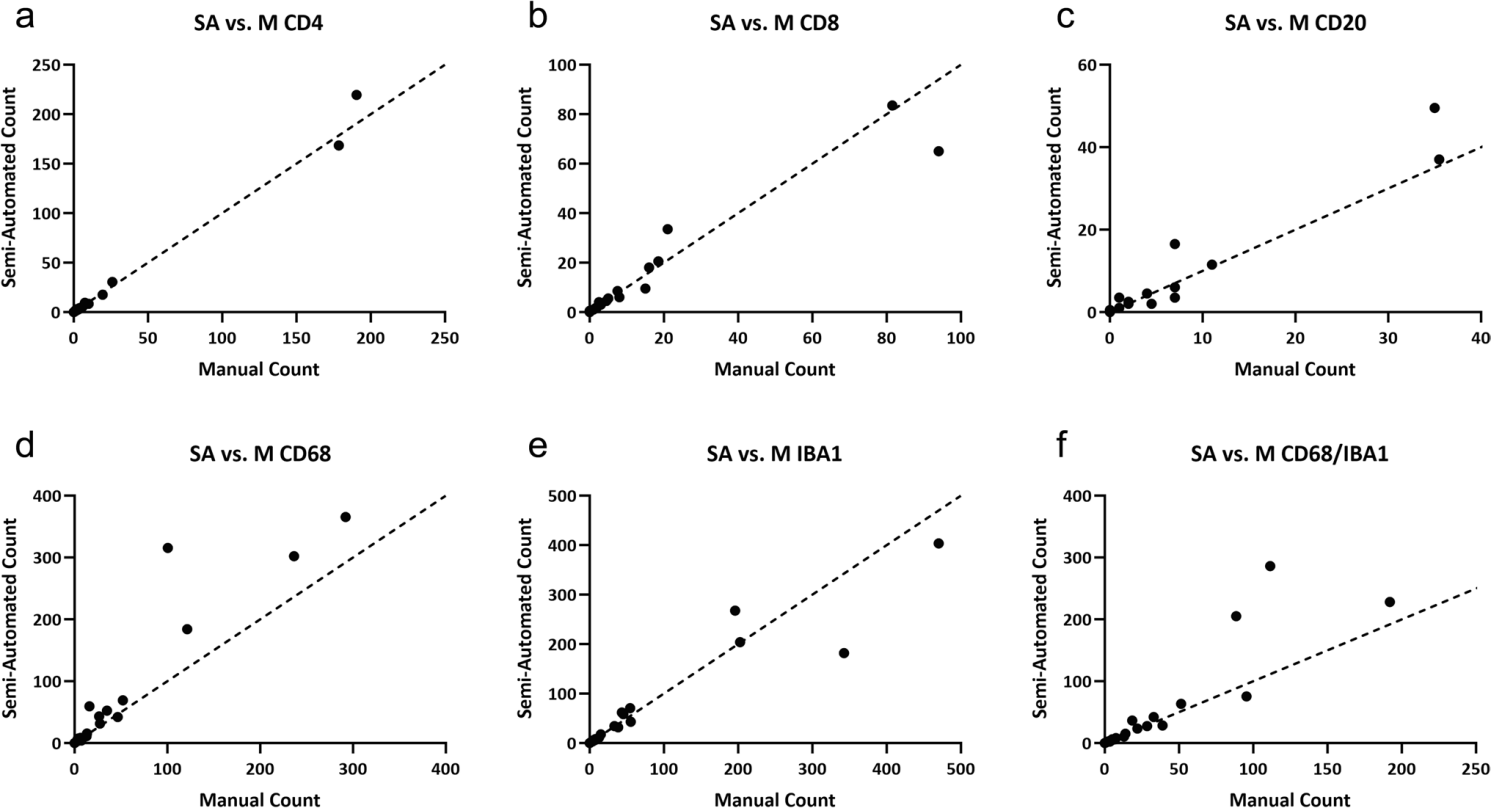
Scatter plots showing the SA vs. M counts for each marker. The dotted lines on each graph represent a line of identity where x = y

**Fig. 7:**
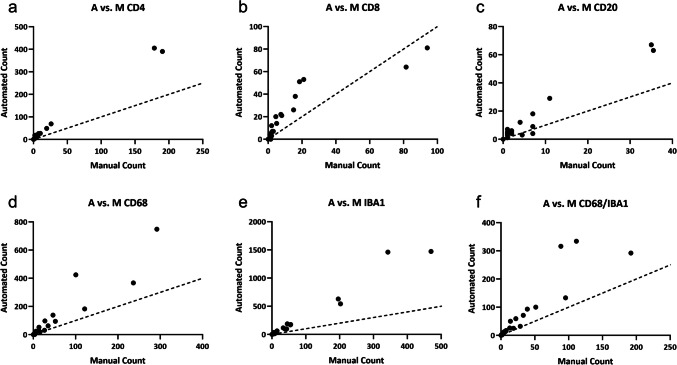
Scatter plots showing the A vs. M counts for each marker. The dotted lines on each graph represent a line of identity where x = y

The semi-automated and automated counts significantly correlated with the manual colocalized cell counts across all cell types and observers (*P* < 0.0001, Table 1). In terms of absolute counts, the semi-automated counts were not significantly different from the manual counts (P > 0.05, Table 2). The automated counts were significantly different from the manual counts (*P* = 0.0042, 0.0458, < 0.0001, 0.0015, 0.0009, 0.0003, Table 2). However, despite the absolute counts differing between automated and manual methods, there was a significantly strong correlation between automated and manual counts (R^2 = 0.9954, 0.776, 0.9601, 0.8829, 0.9680, 0.8210, Table 1). When no colocalized cells were present, the automated method also counted 0 cells. For non-zero cell counts, the automated method consistently counted greater colocalized cell counts than the manual method (manual: 31.66 cells ± 69.05, automated: 82.33 cells ± 210.2) (Fig 6 and 7).

**Table 1:** R^2 & p-values from correlation analysis between SA vs. M & A vs. M counts for each marker

Marker	P-value (Semi-Automated)	P-value (Automated)	R^2 (Semi-Automated)	R^2 (Automated)
CD4	< 0.0001	< 0.0001	0.9889	0.9954
CD8	< 0.0001	< 0.0001	0.9333	0.7764
CD20	< 0.0001	< 0.0001	0.9420	0.9601
CD68	< 0.000001	< 0.0001	0.8776	0.8829
IBA1	< 0.0001	< 0.0001	0.9038	0.9680
CD68/IBA1	< 0.0001	< 0.0001	0.8025	0.8210

**Table 2:** P-values from paired t-test between SA vs. M & A vs. M counts for each marker

Marker	P-value (Semi-Automated)	P-value (Automated)
CD4	0.3609	0.0042
CD8	0.8233	0.0458
CD20	0.2233	< 0.0001
CD68	0.0653	0.0015
IBA1	0.6186	0.0009
CD68/IBA1	0.1322	0.0003

There were no significant differences in measured counts between the two observers, for both the measured and semi-automated counting methods (*P* > 0.05, Table 3).

**Table 3:** Mean overcounting ratios ± standard deviation for each method. The overcounting ratio was calculated as [method count] / [manual count]. The semi-automated method produced an undefined overcounting ratio (0/0) in 28 of 156 samples. The automated method produced an undefined overcounting ratio of (0/0) in the same 28 samples

Marker	Mean Overcounting Ratio (Semi-Automated)	Mean Overcounting Ratio (Automated)
CD4	0.96 ± 0.17	3.2 ± 1.6
CD8	1.1 ± 0.24	2.6 ± 1.4
CD20	1.2 ± 0.66	2.8 ± 1.6
CD68	1.4 ± 0.73	2.3 ± 1.0
IBA1	0.99 ± 0.23	3.3 ± 1.2
CD68/IBA1	1.1 ± 0.49	2.1 ± 0.76

For both easy and hard-to-count morphologies, the automated and semi-automated methods took significantly less time than the manual counting method (*P* < 0.0001). The automated method took significantly less time than the semi-automated method (*P* = 0.0054 for easy-to-count morphologies; *P* < 0.0001 for hard-to-count morphologies) (Table 4).

**Table 4:** P-values from paired t-test between observer 1 vs. observer 2 counts for each marker

Marker	P-value (Manual)	P-value (Semi-Automated)
CD4	0.1857	0.3112
CD8	0.1621	0.7384
CD20	0.6717	0.1626
CD68	0.1913	0.9643
IBA1	0.0809	0.0598
CD68/IBA1	0.5221	0.7360

To evaluate the reproducibility of the semi-automated and automated counting methods under conditions of reduced image quality, we applied these methods to images with a lower signal-to-noise ratio. Using ImageJ, we introduced Gaussian noise (standard deviation = 25) to groups images containing three different cell densities: no cells, a medium density of cells, and a high density of cells. For each density group, six images were selected, each containing one of six different cell types, resulting in a total of 18 analyzed images (*n* = 18). Due to the increased noise, adjustments were made using the “noise-tolerance” tool within the ImageJ plugin, which was set between 50–100 to optimize detection while minimizing false positives (Morelli, et al., 2021).

Statistical analysis did not reveal a significant difference in cell counts obtained from low-quality images compared to high-quality images. On average, semi-automated cell counts from low-quality images were lower than those from high-quality images while automated cell counts were higher (Table 5 and 6).

**Table 5:** Average counting time (s) per image for manual, semi-automated, and automated counting methods. The reported times represent the average duration required to quantify all cells in a single image. Easy-to-count cell types were the CD4, CD8, and CD20 colocalized cells. These cells had round morphologies and were easily distinguishable from each other. Hard-to-count cell types were the CD68, IBA1, and CD68/IBA1 colocalized cells. These cells had more complex morphologies and, therefore, were more difficult to count manually (see Fig. 4)

Marker	Manual	Semi-Automated	Automated
Easy-to-count	90.6	67.5	49.5
Hard-to-count	228.1	130.4	61.6

**Table 6:** Mean cell counts ± standard deviation are presented for images of both high and low quality, analyzed using semi-automated and automated counting methods. P-values were obtained using a paired t-test comparing cell counts from high-quality images to those from low-quality images. "Easy-to-count" morphologies include CD4-, CD8-, and C20-positive cells, while "hard-to-count" morphologies refer to CD68- and IBA1-positive cells

Marker	SA Mean (High Quality Images)	SA Mean (Low Quality Images)	P-Value	Auto. Mean (Low Quality Images)	P-Value
Easy-to-count	40.8 ± 49.4	27.3 ± 33.4	0.055	114.3 ± 186.0	0.15
Hard-to-count	124.3 ± 178.6	99.4 ± 144.5	0.074	222.3 ± 259.8	0.09

These findings suggest that image quality does not significantly impact automated quantification accuracy, but analysis may require noise-tolerance adjustments in samples with a low signal-to-noise ratio.

## Discussion

### Conclusions

In this methods study, we evaluated two different experimental pipelines for streamlining colocalized cell-counting. The semi-automatic method demonstrated high accuracy and efficiency, with its absolute counts showing no significant difference from the gold-standard manual method. While the absolute counts from the fully automatic method differed significantly from manual counts, they exhibited a strong correlation, indicating this method’s reliability in detecting colocalized cells, particularly in situations requiring high-throughput data analysis.

Both the semi-automatic and automatic pipelines were significantly more time efficient than the manual approach, with the automatic method offering near-instantaneous analysis. These pipelines present valuable tools for clinicians and researchers, providing a balance between accuracy and efficiency while significantly reducing the time and resources required for colocalized cell counting.

### Practical Applications

Although the automatic counts differed significantly from manual counts, their strong correlation indicates that they are proportional. This suggests that the automatic cell counting pipeline is a reliable tool for identifying trends or relative changes in colocalized cell populations across various conditions, experiments, or clinical scenarios. In clinical settings, where time is often a limiting factor, this automatic method could be particularly beneficial. It allows clinicians to quickly assess relative differences in cell populations, making it ideal for scenarios such as monitoring disease progression or evaluating therapeutic responses. This method could be especially valuable in high-throughput screening, exploratory research, tracking relative changes over time, and other clinical applications that require timely results rather than precise absolute values.

The semi-automatic method, with its high accuracy and strong correlation with manual counts, is particularly well-suited for applications requiring precise absolute values. This makes it an excellent tool in contexts that demand both reliability and efficiency. Its combination of accuracy and efficiency could streamline workflows in translational research, helping bridge the gap between preclinical findings and clinical applications.

The images analyzed in this study were of exceptionally high quality, with minimal background noise. However, in many research settings involving multiplex immunohistochemistry, image quality can vary significantly. To assess the robustness of our counting methods under lower-quality conditions, we introduced Gaussian noise to a subset of our original images. Analysis of these noisy images did not reveal a significant difference in cell counts compared to the same images without added noise. However, on average the semi-automated method undercounted cells, while the automated method overcounted them. We believe the undercounting in the semi-automated method was likely due to the noise-tolerance filter, which may have suppressed true signal detection to reduce false positives. In contrast, the automated method, using the CellProfiler pipeline, analyzed unprocessed images without any noise reduction preprocessing, leading to overcounting. These findings suggest that the automated method is most suitable for higher-quality images with a high signal-to-noise ratio, whereas lower-quality images may require preprocessing or the use of a semi-automated approach with optimized noise filtering. Researchers should carefully consider image quality when selecting a counting method to ensure accurate quantification.

Although the automated method may be useful in certain scenarios, it is important to acknowledge the potential consequences of its consistent overcounting of colocalized objects by a factor of 2-3x. This systematic overestimation introduces a reproducible yet inaccurate bias, potentially leading to false positives in the dataset by counting non-colocalized objects. As a result, the method's specificity is low. In clinical research settings, automated counting may produce results that are not directly comparable to manual counts or other established data analysis methods. This discrepancy can make it difficult to interpret findings and draw meaningful conclusions, particularly when comparing results across studies.

To mitigate these issues, automated counts should be regularly validated against manual counts. If the overcounting bias is consistent, researchers may be able to apply a correction factor to adjust the dataset. However, this approach assumes that the bias remains stable across different conditions, which is not always the case. As demonstrated in this study, the degree of overcounting varied depending on cell type, highlighting the need for careful validation in each experimental context.

As artificial intelligence and deep learning techniques continue to evolve, the future of counting colocalized cells, particularly those with varying morphologies, looks promising. Innovations in methodologies like ColocML and DeepCOLOR highlight the potential for enhanced accuracy and efficiency in analyzing complex cellular interactions. ColocML, for example, utilizes a gold-standard dataset informed by expert annotations to train machine learning algorithms that quantify colocalized structures in mass spectrometry samples (Ovchinnikova et al., 2020). Meanwhile, DeepCOLOR integrates single-cell transcriptomics and spatial transcriptomics, enabling researchers to visualize and understand gene expression dynamics within tissue environments (Kojima, et al., 2024). Colocalization methods could incorporate sophisticated AI training like this to not only count colocalized cells with greater precision but also differentiate between various cell types based on their unique morphological characteristics. This progress could pave the way for deeper insights into cellular functions and interactions in diverse biological contexts, enhancing our understanding of complex biochemical processes.

### Limitations

The tissue samples analyzed in this study underwent multiplex-immunohistochemical (MP-IHC) staining for proteins of interest. It would be of particular interest to explore how these methods perform when applied to counting colocalized signals from another common tissue staining technique, RNAscope, as the expression patterns in this technique are typically more granular and dot-like compared to the more uniform expression patterns seen with protein markers. RNAscope staining detects mRNA molecules, producing punctate signals in the cytoplasm or nucleus of cells, which could present a unique challenge for cell counting methods like those presented here, that are optimized for more uniform, structured markers. Given that these methods have shown success with well-defined cell morphologies and protein markers, it would be valuable to investigate their adaptability to the more complex and dispersed signals seen in RNAscope.

Additionally, the fluorescent MP-IHC performed in this study was of exceptionally high quality, with minimal background noise. As described earlier, segmentation is the most challenging step in object-based colocalization analysis. Because our multi-fluorescent images were such high quality, segmentation was a relatively smooth process in our OBCA pipeline since cell signal intensity and morphology was very distinguishable from the background noise (especially for our easy-to-count cell morphologies). However, in situations where researchers may not have access to such high-quality staining techniques, higher levels of background noise could pose challenges for obtaining accurate results. In cases where samples exhibit a lower signal-to-noise ratio, users may choose to rely on the semi-automatic method, as it allows for greater manual adjustments to account for noise and improve accuracy. This highlights the importance of considering sample quality and noise levels when selecting the most appropriate method for colocalized cell counting.

Both the semi-automatic and automatic methods demonstrated their robustness by successfully quantifying colocalized markers in cells with diverse morphologies, such as macrophages labeled with IBA1, CD68, and DAPI. This versatility suggests their potential applicability in analyzing additional colocalized structures that deviate from the circular morphology of typical lymphocytes included in this study. For instance, these methods could be applied to examine the colocalization of neurotransmitter receptors with the neurons that produce those neurotransmitters in specific brain regions, as well as the colocalization of multiple neurotransmitters and pre- and post-synaptic structures.

Applying these techniques to the study of neurotransmitter interactions could provide deeper insight into their functional relationships. For example, previous research has demonstrated that Substance P (SP) colocalizes with classical neurotransmitters in the central nucleus of the amygdala, a region implicated in neuropsychiatric disorders. Expanding our colocalization methods to investigate such interactions could provide valuable information on the molecular underpinnings of these conditions. Additionally, research on VGLUT1 and VGLUT2 has highlighted the transient colocalization of these transporters in developing cortical circuits, emphasizing the importance of colocalization in synaptic maturation (Zinchuk & Grossenbacher-Zinchuk, 2009). Adapting our methods to study similar dynamic changes in neurotransmitter systems could enhance our understanding of synaptic plasticity and its role in both normal development and disease states. Moreover, leveraging quantitative colocalization to examine alterations in neurotransmitter systems in neurodegenerative diseases or in response to pharmacological treatments would allow for more precise quantification of disease progression and therapeutic effectiveness. By leveraging these tools, semi-automatic and automatic quantification methods could efficiently and accurately contribute to a more comprehensive understanding of the complex regulatory processes within the nervous system.

While these methods proved capable of quantifying colocalized structures with diverse morphologies, challenges arose with cells that deviated from standard circular shapes. For example, IBA1 and CD68 positive macrophages, with their irregular and often fragmented morphologies, required additional manual correction by users to account for overcounting, as illustrated in Fig. 4. This correction process significantly increased the time required for manual and semi-automated methods, particularly when analyzing multiple samples. The added time and complexity associated with these morphologies highlights the importance of selecting the appropriate method based on the type of cells and desired study parameters. Fully automated approaches may mitigate this limitation, although further refinements in their algorithms may be necessary to achieve greater precision for complex morphologies.

Beyond cell morphology, these methods could potentially be applied to other imaging modalities, such as brightfield images. Brightfield imaging, commonly used in histological studies and clinical settings, includes techniques like hematoxylin and eosin staining or standard DAB and nickel-enhanced DAB staining. Expanding the application of these methods to such modalities could significantly enhance their utility, particularly for pathologists and researchers working with non-fluorescent samples. Investigating the performance of these pipelines across diverse imaging techniques and cellular morphologies would provide a more comprehensive understanding of their capabilities and limitations.

## Supplementary Information

Below is the link to the electronic supplementary material.

Supplementary file1 (XLSX 20 KB)

## Data Availability

No datasets were generated or analysed during the current study.

## References

[CR1] Barlow, A. L., Macleod, A., Noppen, S., Sanderson, J., & Guerin, C. J. (2010). Colocalization analysis in fluorescence micrographs: Verification of a more accurate calculation of pearson’s correlation coefficient. *Microscopy and Microanalysis,**16*(6), 710–724. 10.1017/S143192761009389X 20946701 10.1017/S143192761009389X

[CR2] Bolte, S., & Cordelieres, F. P. (2006). A guided tour into subcellular colocalization analysis in light microscopy. *Journal of Microscopy,**224*(Pt 3), 213–232. 10.1111/j.1365-2818.2006.01706.x 17210054 10.1111/j.1365-2818.2006.01706.x

[CR3] Fonseca, A., & Rosa, S. (2025). Detection and Automated Quantification of Nucleocytoplasmic RNA Fractions in Arabidopsis Using smFISH. *Methods in Molecular Biology,**2873*, 187–203. 10.1007/978-1-0716-4228-3_11 39576603 10.1007/978-1-0716-4228-3_11

[CR4] Gilles, J. F., Dos Santos, M., Boudier, T., Bolte, S., & Heck, N. (2017). DiAna, an ImageJ tool for object-based 3D co-localization and distance analysis. *Methods,**115*, 55–64. 10.1016/j.ymeth.2016.11.016 27890650 10.1016/j.ymeth.2016.11.016

[CR5] Jones, T. R., et al. (2009). Scoring diverse cellular morphologies in image-based screens with iterative feedback and machine learning. *Proceedings of the National Academy of Sciences,**106*(6), 1826–1831. 10.1073/pnas.0808843106 10.1073/pnas.0808843106 PMC2634799 19188593

[CR6] Kojima, Y., Mii, S., Hayashi, S., Hirose, H., Ishikawa, M., Akiyama, M., & Shimamura, T. (2024). Single-cell colocalization analysis using a deep generative model. *Cell Systems,**15*(2), 180–192. 10.1016/j.cels.2024.01.007 38387441 10.1016/j.cels.2024.01.007

[CR7] Lunde, A., & Glover, J. C. (2020). A versatile toolbox for semi-automatic cell-by-cell object-based colocalization analysis. *Scientific Reports,**10*(1), 19027. 10.1038/s41598-020-75835-7 33149236 10.1038/s41598-020-75835-7 PMC7643144

[CR8] Manders, E. M. M., Verbeek, F. J., & Aten, J. A. (1993). Measurement of co-localization of objects in dual-colour confocal images. *Journal of Microscopy,**169*(3), 375–382. 10.1111/j.1365-2818.1993.tb03313.x 33930978 10.1111/j.1365-2818.1993.tb03313.x

[CR9] Meijering, E. (2012). Cell segmentation: 50 years down the road [life sciences]. *IEEE Signal Processing Magazine,**29*(5), 140–145. 10.1109/MSP.2012.2204190

[CR10] Morelli, R., Clissa, L., Amici, R., Cerri, M., Hitrec, T., Luppi, M., & Zoccoli, A. (2021). Automating cell counting in fluorescent microscopy through deep learning with c-ResUnet. *Scientific Reports,**11*(1), 22920. 10.1038/s41598-021-01929-5 34824294 10.1038/s41598-021-01929-5 PMC8617067

[CR11] Ovchinnikova, K., Stuart, L., Rakhlin, A., Nikolenko, S., & Alexandrov, T. (2020). ColocML: Machine learning quantifies co-localization between mass spectrometry images. *Bioinformatics,**36*(10), 3215–3224. 10.1093/bioinformatics/btaa085 32049317 10.1093/bioinformatics/btaa085 PMC7214035

[CR12] Rajani, S., Gell, C., Abakir, A., & Markus, R. (2021). Computational Analysis of DNA Modifications in Confocal Images. *Methods in Molecular Biology,**2198*, 227–254. 10.1007/978-1-0716-0876-0_19 32822036 10.1007/978-1-0716-0876-0_19

[CR13] Riggle, B. A., et al. (2020). CD8+ T cells target cerebrovasculature in children with cerebral malaria. *The Journal of Clinical Investigation,**130*(3), 1128–1138. 10.1172/JCI133474 31821175 10.1172/JCI133474 PMC7269583

[CR14] Zhang, H., et al. (2018). Quantitative image analysis of protein expression and colocalisation in skin sections. *Experimental Dermatology,**27*(2), 196–199. 10.1111/exd.13457 29094393 10.1111/exd.13457

[CR15] Zinchuk, V., & Grossenbacher-Zinchuk, O. (2009). Recent advances in quantitative colocalization analysis: Focus on neuroscience. *Progress in Histochemistry and Cytochemistry,**44*(3), 125–172. 10.1016/j.proghi.2009.03.001 19822255 10.1016/j.proghi.2009.03.001

